# Neutrophil-Derived IL-17 Promotes Ventilator-Induced Lung Injury *via* p38 MAPK/MCP-1 Pathway Activation

**DOI:** 10.3389/fimmu.2021.768813

**Published:** 2021-12-15

**Authors:** Xiaoting Liao, Weikang Zhang, Huijun Dai, Ren Jing, Mengling Ye, Wanyun Ge, Shenglin Pei, Linghui Pan

**Affiliations:** ^1^ Department of Anesthesiology, Guangxi Key Laboratory of Basic Research on Perioperative Organ Function Injury & Control, and Guangxi Medical Engineering Research Center of Tissue Injury and Repair, Guangxi Medical University Cancer Hospital, Nanning, China; ^2^ Department of Anesthesiology, The Cancer Hospital of the University of Chinese Academy of Sciences (Zhejiang Cancer Hospital), Hangzhou, China

**Keywords:** ventilator-induced lung injury, IL-17, inflammatory response, p38 MAPK, MCP-1

## Abstract

Ventilator-induced lung injury (VILI) is one of the most common complications of mechanical ventilation and can severely affect health. VILI appears to involve excessive inflammatory responses, but its pathogenesis has not yet been clarified. Since interleukin-17 (IL-17) plays a critical role in the immune system and the development of infectious and inflammatory diseases, we investigated here whether it plays a role in VILI. In a mouse model of VILI, mechanical ventilation with high tidal volume promoted the accumulation of lung neutrophils, leading to increased IL-17 levels in the lung, which in turn upregulated macrophage chemoattractant protein-1 *via* p38 mitogen-activated protein kinase. Depletion of neutrophils decreases the production IL-17 in mice and inhibition of IL-17 significantly reduced HTV-induced lung injury and inflammatory response. These results were confirmed *in vitro* using RAW264.7 macrophage cultures. Our results suggest that IL-17 plays a pro-inflammatory role in VILI and could serve as a new target for its treatment.

## Introduction

Mechanical ventilation is widely used to support patients with acute respiratory failure and other severe diseases. However, it is associated with ventilator-induced lung injury (VILI), a syndrome that can cause or exacerbate lung injury through volu-, baro- and biotrauma (1–3). VILI can lead to overwhelming inflammatory responses, such as activation of the innate immune system and release of inflammatory molecules, which increase alveolar edema and pulmonary vascular permeability (4). We previously found that alveolar macrophages (AM) played an important role in promoting inflammation in the occurrence and development of VILI-mediated lung inflammation (5). VILI-induced inflammation and the release of inflammatory factors appear to involve phosphorylation of p38 mitogen-activated protein kinase (MAPK) (6), but many details of the processes behind VILI remain unclear.

Interleukin (IL)-17, a pro-inflammatory cytokine that was first detected in human peripheral blood (7, 8), contributes to inflammatory, tumor, and autoimmune diseases (9), as it can induce the production of chemokines and inflammatory molecules such as tumor necrosis factor-α, thereby activating innate immune responses and recruiting immunecells (10). Increased IL-17 levels in the serum, sputum, and broncho alveolar lavage fluid (BALF) of asthmatic patients positively correlate with disease severity (11–13). Moreover, IL-17 was found to aggravate lung inflammation and reduce lung function during H1N1 influenza virus infection and *Klebsiella pneumoniae*-induced pneumonia (14, 15).

The p38 MAPK, a member of the MAPK superfamily, is activated by various pro-inflammatory and stressful stimuli, mediating the release of inflammatory cytokines involved in the regulation of various diseases (16–19). Macrophage chemoattractant protein-1 (MCP-1), also known as C–C chemokine ligand 2 (CCL2), is an important downstream molecule of p38 MAPK activation that modulates the recruitment of inflammatory cells into damaged organs and tissues (20). MCP-1 may also exacerbate lung injury in patients with polycystic kidney disease and acute respiratory distress syndrome (21, 22).

In this study, we aimed to determine whether IL-17 plays a role in VILI. In particular, we asked whether IL-17 influences VILI by activating p38 MAPK/MCP-1 signaling.

## Materials and Methods

### Animals

Male C57BL/6 mice aged 6–8 weeks with a body weight of 22 ± 5 g were purchased from the Experimental Animal Center of Guangxi Medical University. All mice were raised at 20–25°C and 30–70% relative humidity and provided with protein-containing feed and purified water. The study was approved by the Experimental Animal Committee of Guangxi Medical University, and all animal experiments were performed in accordance with local and international ethical guidelines. Age- and weight-matched controls were used in all experiments.

### Mouse Model of VILI

Before tracheal incubation, all mice were fasted for 8h and left without water for 4 h. Mice were then anesthetized by intraperitoneal injection of sodium pentobarbital (60 mg/kg); they were administered in 1/3 of the first dose to maintain anesthesia every 45 min. A sterile 20G intravenous trocar was used for tracheal intubation at a depth of 1.2 ± 0.5 cm. The catheter was fixed in the trachea to prevent one-lung ventilation and was connected to a small-animal ventilator (catalog no. SAR-1000, CWE) for mechanical ventilation with air. Non-ventilated mice were used as controls, while mechanically ventilated mice were ventilated with either normal tidal volume (NTV, 7 mL/kg) or high tidal volume (HTV, 20 mL/kg).

To explore the source of IL-17 in the mice treated with HTV, the mice were injected intraperitoneally with anti-Ly6G antibody (400 µg; BP0075, BioXCell) one day before ventilation to deplete neutrophils *in vivo*. To assess the role of IL-17 in the p38 MAPK/MCP-1 pathway after HTV ventilation, mice were randomly divided into four groups (H1-4) depending on whether ventilation lasted 1, 2, 3, or 4 h. One day before ventilation, the mice were injected intraperitoneally with anti-IL-17 antibody (400 µg; BE0173, BioXCell), and at 30 min before anesthesia 200 µg anti-IL-17 antibody was administered through intratracheal instillation. Recombinant mouse IL-17 (rmIL-17; 0.5 μg, catalog no. 576006, Biolegend) was administered through intratracheal instillation 30 min before anesthesia. HTV-ventilated mice administered with saline served as controls.

### Cell Strains

RAW264.7 mononuclear macrophage leukemia cells were purchased from the American Type Culture Collection. Cells were grown in Dulbecco’s Modified Eagle’s medium (DMEM; catalog no. C11995500BT, Gibco), and the optimal infection conditions and multiplicity of infection were determined. In the optimized procedure, a 500 μL suspension of RAW246.7 cells and 20 μL of HitransG A infection-enhancing solution were added to each well of a 24-cell well plate. After cells reached 50% confluence, they were co-cultured with 20 μL of HitransG P infection-enhancing solution and lentivirus infection solution. After 16 h, the medium was replaced with fresh DMEM medium. At 72 h after transfection, the transfection efficiency was determined by fluorescence microscopy. Transfected cells were selected using 2 μg/mL puromycin, which was reduced to 1 μg/mL for further screening.

To clarify the mechanism of IL-17 *in vitro*, wild-type RAW264.7 cells were stimulated with rmIL-17(100 ng/mL) and cultured for 1, 2, 3, 4, or 5 h. Non-stimulated wild-type RAW264.7 cells served as controls. RAW264.7 cells transfected with negative-control lentivirus and stimulated with rmIL-17 for 4 h are referred to below as the CN group. RAW264.7 cells transfected with lentivirus encoding short hairpin RNA (shRNA) against p38 MAPK (Shanghai Jikai Gene Technology, Shanghai, China) and cultured with rmIL-17 for 4 h are referred to below as the shRNA group.

### Inflammatory Response

After the animal model was established, all mice were sacrificed by carotid artery bleeding, and their lungs were excised and weighed immediately (wet weight) and again after drying in an oven at 60°C for 72 h (dry weight). All operations were conducted carefully to minimize the risk of inflammatory activation.

The total protein concentration in BALF was estimated using the bicinchoninic acid (BCA) assay (P0012S, Beyotime) to assess pulmonary permeability. The levels of IL-17 and MCP-1were assayed in BALF and culture medium were detected using the ELISA kits (CSB-E04608m, Cusabio Biotech and EK0568, Boster) according to the manufacturers’ instructions.

### Histopathological Analysis

The pathological damage in mouse lungs after HTV ventilation was assessed after hematoxylin–eosin (H&E) staining. Briefly, the collected mouse lungs were fixed with 3.7% paraformaldehyde, embedded in paraffin, and cut into 5-μm sections. After dewaxing, the sections were stained and the tissue morphology was observed with a microscope. The degree of lung injury was evaluated by applying a four-point scale (0, no injury; 1, minor injury; 2, moderate injury; 3, severe injury) to each of the following parameters (6, 23, 24): alveolar congestion, hemorrhage, inflammatory cell infiltration, and alveolar wall thickening. These individual scores were summed to give the total score of lung damage.

### Immunoblot Analysis

Lung tissues and RAW264.7 cells were lysed in RIPA lysis buffer (P0013B,Beyotime) supplemented with protease inhibitor (ST505, Beyotime) and phosphatase inhibitor (P1050, Beyotime), and the protein concentration in each sample was determined using the BCA assay. Samples were denatured, fractionated by sodium dodecyl sulfate-polyacrylamide gel electrophoresis, and transferred to polyvinylidene difluoride membranes. The non-specific membrane binding sites were blocked with 5% bovine serum albumin (BSA) for 1 h. Membranes were then incubated at 4°C overnight with primary antibodies (all diluted 1:1000) against the following proteins: IL-17 (ab79056, Abcam), MCP-1 (catalog no. 2029, CST),p38 MAPK (catalog no. 9212, CST), and phosphorylated (p)-p38 MAPK (catalog no. 4511, CST). Blots were washed several times with PBS buffer, then incubated for 1 h with goat anti-rabbit IgG secondary antibody (diluted 1:15000; ab96899, Abcam). The bands were visualized using a fluorescent scanner. As an internal reference, GAPDH was immunostained using an antibody (1:1000; catalog no. 5174, CST).

### Quantitative Real-Time Polymerase Chain Reaction (qRT-PCR)

Total RNA was isolated from lungs and RAW264.7 cells using the MiniBEST Universal RNA Extraction kit (catalog no. 9767, TaKaRa Bio, Otsu) following the manufacturer’s instructions. After determining RNA quality and quantity by spectrophotometry, cDNA was synthesized using the PrimeScript™ RT Master Mix kit(RR036A, TaKaRa) and amplified with the SYBR^®^ Premix Ex Taq™ II(TliRNaseH Plus) kit(RR820A, TaKaRa). The following primers were used: GAPDH forward, 5′-TGTGTCCGTCGTGGATCTGA-3′; GAPDH reverse,5′-TTGCTGTTGAAGTCGCAGGAG-3′; MCP-1 forward, 5′-CAGGTCCCTGTCATGCTTCT-3′; MCP-1 reverse, 5′-GTGGGGCGTTAACTGCATCT-3′; p38 forward, 5′-CTGTCGAGACCGTTTCAGTCCA-3′; p38 reverse,5′-GTGTGAACACATCCAACAGACCAA-3′. The relative expression of GAPDH, MCP-1, and p38 was determined using the 2^−ΔΔ^Ct method and normalized to expression of GAPDH.

### Flow Cytometry

Flow cytometry was used to detect the accumulation of neutrophils in the lung tissues of mice and the expression of IL-17 in neutrophils, Th17 cells, and T cells. Lung sections were treated with 200 U/mL Type I collagenase (950 μL, 17018029, Gibco), DNaseII(20 U,4942078001, Merck), and FBS (50 μL,10099141, Gibco)to prepare single-cell suspensions. Red blood cell lysis solution (1mL) was then added to each cell suspension to remove red blood cells and adjust the density to 5 × 10^6^ mL^−1^. Cells were labelled using antibodies against Ly6G (catalog no.REA256, Miltenyi Biotec), CD4 (catalog no. GK1.5, Miltenyi Biotec), and CD90 (catalog no. 105201, Biolegend). After washing with PBST buffer, cells were ruptured and intracellular proteins were labelled for 20 min using anti-IL-17 antibody (catalog no. 560522, BD Horizon) and analyzed at 4 °C using a flow cytometer.

### Immunofluorescence

Immunofluorescence was used to assess the expression of MCP-1 in RAW264.7 cells; the expression of IL-17 (catalog no.14-7179-82, Thermo Fisher Scientific), CD4 (catalog no.100401, Biolegend), CD90 (catalog no.105201, Biolegend), and Ly6G (catalog no.127601, Biolegend) in mouse lungs; and the source of IL-17 in mouse lung tissues. All sections were fixed with 3.7% paraformaldehyde, ruptured with 0.2% Triton X-100, and blocked with BSA and goat serum. After incubation with the above- mentioned primary antibodies at 4°C overnight, samples were incubated with goat anti-rabbit IgG secondary antibody for 1 h. Nuclei were stained with 4′,6-diamidino-2-phenylindole, then samples were observed using a confocal laser scanning microscope.

### Statistical Analysis

Statistical analysis was performed using SPSS 22.0 software (IBM, USA). All data were reported as mean ± standard deviation (SD). Differences in parametric data that showed a normal distribution were assessed for significance using one-way analysis of variance and the Bonferroni test. Differences associated with a two-tailed *P* < 0.05 were considered statistically significant.

## Results

### HTV Ventilation Upregulates IL-17 in Lung Tissues

**Figure 1:**
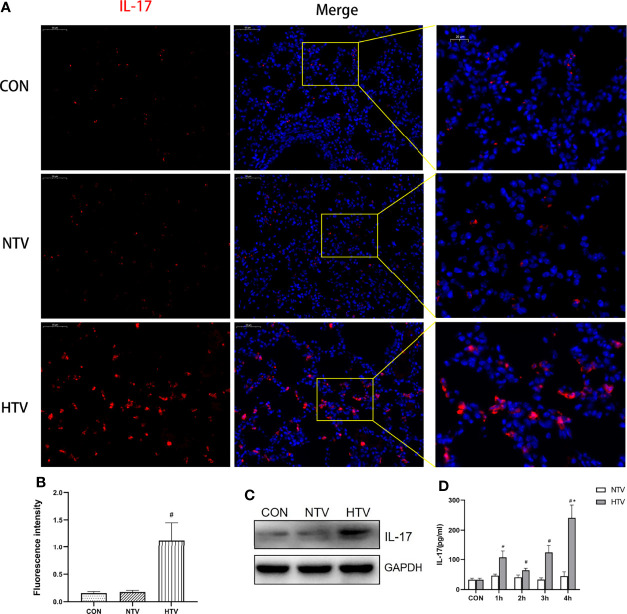
Ventilation with high tidal volume (HTV) up regulates interleukin (IL)-17 in the lungs. **(A–C)** IL-17 expression in the lung tissue of mice ventilated for 4 h with normal tidal volume (NTV, 7 mL/kg) or high tidal volume (HTV, 20 mL/kg). **(D)** Concentration of IL-17 in bronchoalveolar lavage fluid (BALF) of mice ventilated with NTV and HTV for 1 h, 2 h, 3 h, or 4 h. CON, non-ventilated mice. ^#^ *P* < 0.05 *vs*. CON group; ^*^ *P* < 0.05 *vs*. H1, H2, H3, or H4 group.

To assess the effect of HTV ventilation on IL-17 expression, we determined the levels of IL-17 in non-ventilated, NTV, and HTV ventilated mice. The IL-17 levels in lungs were significantly higher in the HTV group than in the control and NTV groups (**Figures 1A–C**). Compared with CON group, the concentration of IL-17 in BALF also increased in HTV group (**Figure 1D**). These results suggest that IL-17 is closely associated with the development of VILI.

### IL-17 Is Mainly Produced by Lung Neutrophils After HTV Ventilation

**Figure 2:**
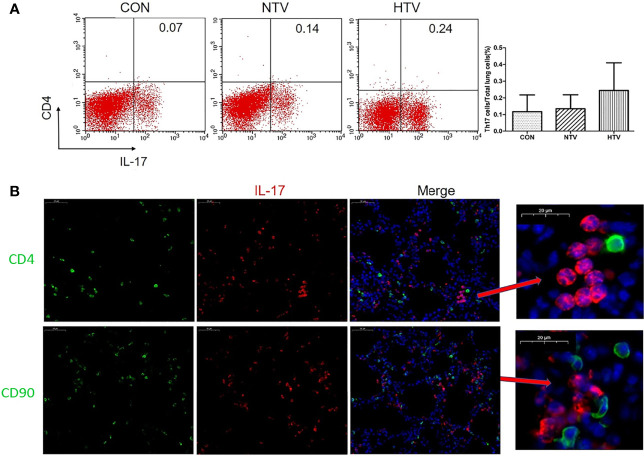
Interleukin (IL)-17 does not originate from Th17 cells and T cells after high tidal volume (HTV) ventilation. **(A)** Number of CD4^+^IL-17^+^ cells in mouse lung tissue. **(B)** Co-localization of IL-17 with CD4 and CD90 in the lung tissues. Data are shown as mean ± SD (n = 6). CON, non-ventilated mice; NTV, ventilated for 4 h with normal tidal volume; HTV, ventilated for 4 h with high tidal volume.

**Figure 3:**
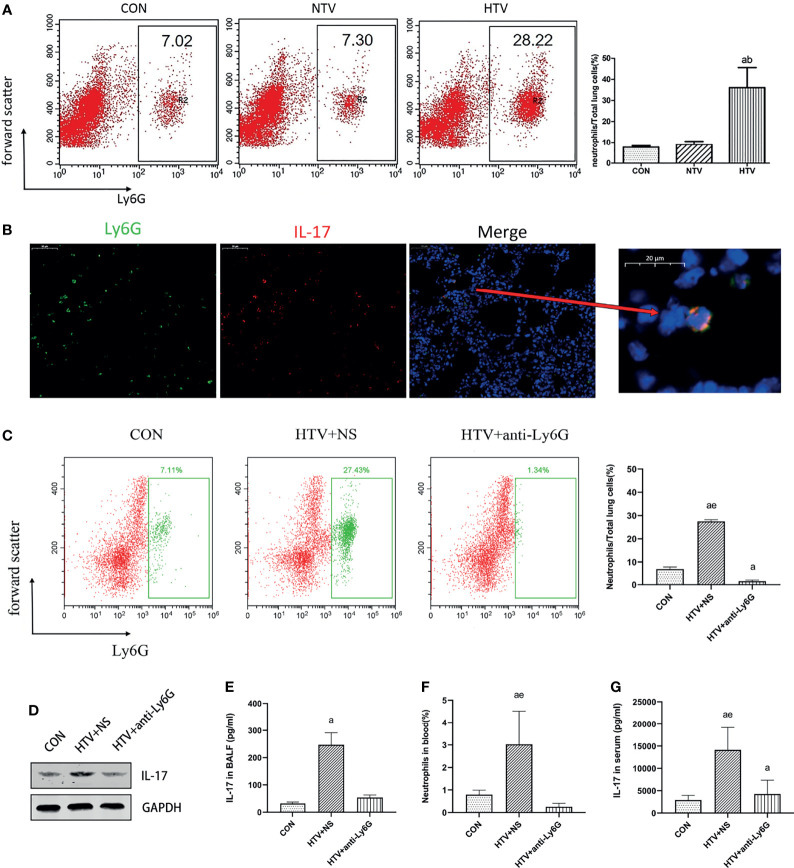
Interleukin (IL)-17 is produced by lung neutrophils after HTV. **(A)** Aggregation of neutrophils in mouse lung tissue. **(B)** Co-localization of IL-17 with Ly6G in the lung tissues. **(C)** Neutrophils in mouse lung cells. **(D)** IL-17 levels in mouse lung tissues. **(E)** Concentration of IL-17 in BALF. **(F)** Neutrophils in blood of mice. **(G)** Concentration of IL-17 in serum. Data are shown as mean ± SD (n = 6). ^a^ *P* < 0.05 *vs*. CON group, ^b^P < 0.05 vs. NTV group, ^e^ *P* < 0.05 *vs*. HTV+anti-Ly6G group. CON, non-ventilated mice; HTV+NS, intraperitoneal injection with normal saline (NS) and ventilated for 4 h with high tidal volume; HTV+anti-Ly6G, intraperitoneal injection with anti-Ly6G antibody and ventilated for 4 h with high tidal volume.

IL-17 is expressed mainly by Th17 cells and neutrophils (25, 26). Flow cytometry showed no significant difference in the numbers of CD4^+^IL-17^+^ cells among control, NTV and HTV groups (**Figure 2A**). IL-17 in the HTV group did not co-localize with the Th17 cell surface marker CD4 or the T cell surface marker CD90 (**Figure 2B**), suggesting that IL-17 is not produced by Th17 cells and T cells during VILI. In contrast, the number of Ly6G^+^neutrophils was significantly higher in the HTV group than in the other two groups (**Figure 3A**). Immunofluorescence shows that IL-17 co-localizes with the neutrophils surface marker Ly6G (**Figure 3B**). Furthermore, when neutrophils were depleted in mice (**Figures 3C**, **F**), compared with the HTV group, the levels of IL-17 in lung tissue (**Figure 3D**), BALF (**Figure 3E**) and plasma (**Figure 3G**) were significantly reduced in the HTV+anti-Ly6G group. These results indicate that IL-17 is produced mainly by Ly6G^+^neutrophils that are recruited to the lungs at the onset of VILI.

### IL-17 Inhibition Attenuates VILI-Induced Inflammation

**Figure 4:**
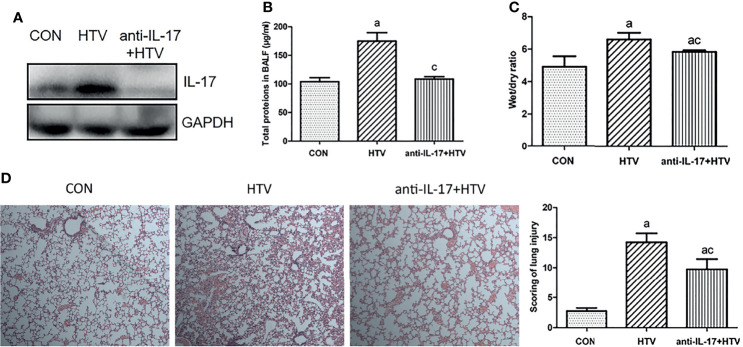
Inhibition of interleukin (IL)-17 attenuates ventilator-induced pulmonary edema. **(A)** IL-17 levels in mouse lung tissues. **(B)** Total protein concentration in bronchoalveolar lavage fluid (BALF) of mice. **(C)** Wet weight to dry weight (W/D) ratio of mouse lungs. **(D)** Histopathological analysis and degree of lung injury after ventilation with high tidal volume (HTV). Magnification, 400×. Data are shown as mean ± SD (n = 6). ^a^ *P* < 0.05 *vs*. CON group, ^c^ *P* < 0.05 *vs*. HTV group. CON, non-ventilated mice; HTV, ventilated for 4 h with high tidal volume; anti-IL-17+HTV, treated with anti-IL-17 antibody and ventilated for 4 h with high tidal volume.

The wet weight to dry weight ratio (W/D) of mouse lungs reflects the degree of lung edema, as the fluid that accumulates in the lung interstitium, alveolar cavity, and small bronchi increases lung tissue wet weight. Moreover, lung inflammation leads to the secretion of several inflammatory molecules in the alveolar cavity. We took advantage of these phenomena to assess whether inhibiting the function of IL-17 using an anti-IL-17 antibody (**Figure 4A**) would reduce pulmonary edema and total protein levels in BALF. Indeed, treating the HTV group with anti-IL-17 antibody led to significantly lower pulmonary edema and total BALF protein (**Figures 4B, C**), as well as milder alveolar congestion, alveolar interstitial thickening, and neutrophil infiltration (**Figure 4D**). These results suggest that blocking IL-17 can mitigate inflammation caused by VILI.

### rmIL-17 Upregulates MCP-1 in RAW264.7 Cells *via* the p38 MAPK Pathway

**Figure 5:**
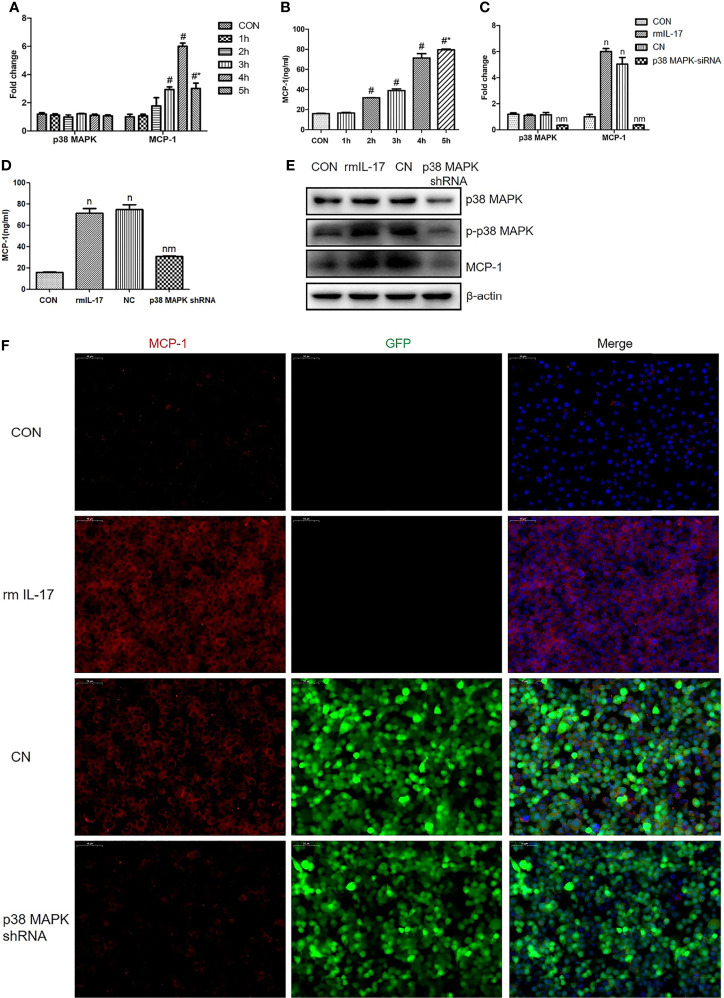
Recombinant mouse interleukin-17 (rmIL-17) upregulates MCP-1 in RAW264.7 cells *via* the p38 MAPK pathway. **(A, C)** Levels of mRNAs encoding p38 and MCP-1 in RAW264.7 cells. **(B, D)** Concentration of MCP-1 in medium from RAW264.7 cultures. **(E)** Levels of p38, phosphorylated (p)-p38, and MCP-1 in RAW264.7 cells. **(F)** Expression of MCP-1 in RAW264.7 cells, as determined by immunofluorescence. Data are shown as mean ± SD (n = 4). ^#^ *P* < 0.05 *vs*. CON group, ^*^ *P *< 0.05 *vs*. wild-type RAW264.7 cells cultured with rmIL-17, ^n^ *P* < 0.05 *vs.* CON group, ^m^ *P* < 0.05 *vs*. CN group. CON, wild-type RAW264.7 cells; 1h, 2h, 3h, 4h, wild-type RAW264.7 cells stimulated with rmIL-17 (100 ng/mL) and cultured for 1, 2, 3, 4, or 5 h; rmIL-17, wild-type RAW264.7 cells cultured with rmIL-17 (100 ng/mL); CN, negative lentivirus-transfected RAW264.7 cells stimulated with rmIL-17 (100 ng/mL) for 4 h; p38 MAPK shRNA, RAW264.7 cells transfected with lentivirus encoding short hairpin RNA against p38 MAPK and cultured with rmIL-17 (100 ng/mL) for 4 h.

The p38 MAPK, as a classic downstream molecule of TLR4, has been associated with the pathogenesis of VILI (6). RAW264.7 cells were stimulated with rmIL-17 to examine whether IL-17 may interact with p38 MAPK. RT-qPCR analysis revealed that rmIL-17 significantly upregulatedMCP-1 mRNA expression (**Figures 5A, B**), which p38 MAPK knockdown partially reversed (**Figures 5C–F**). Western blot and RT-qPCR analysis also showed that rmIL-17 promoted the phosphorylation of p38 MAPK and MCP-1, without affecting total levelsofp38-MAPK (**Figures 5A, E**). These results suggest that IL-17 upregulates the expression ofMCP-1 by promoting the phosphorylation of p38 MAPK in lung macrophages.

### IL-17 Upregulates MCP-1 in Mouse Lung Tissues *via* the p38 MAPK Pathway

**Figure 6:**
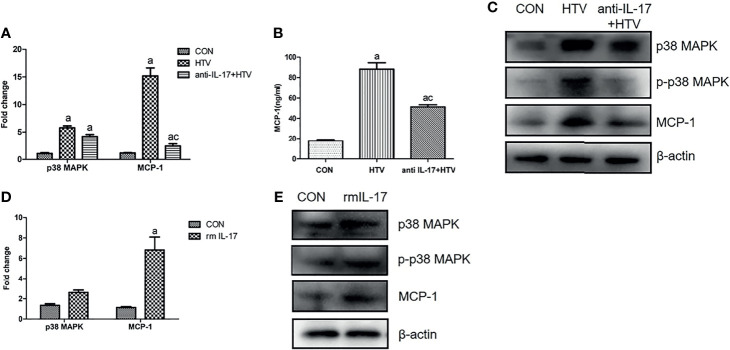
Interleukin (IL)-17 upregulates MCP-1 in mouse lung tissues *via* the p38 MAPK pathway. **(A, D)** Levels of mRNAs encoding p38 and MCP-1 in mouse lung tissues. **(B)** Concentration of MCP-1 in bronchoalveolar lavage fluid of mice. **(C, E)** Expression of p38 MAPK, phosphorylated (p)-p38 MAPK, and MCP-1 in mouse lung tissues. Data are shown as mean ± SD (n = 6). ^a^ *P* < 0.05 *vs*. CON group, ^c^ *P* < 0.05 *vs*. HTV. CON, non-ventilated mice; HTV, ventilated for 4 h with high tidal volume; anti-IL-17+HTV, treated with anti-IL-17 antibody and ventilated for 4 h with high tidal volume; rmIL-17+HTV, treated with rmIL-17 and ventilated for 4 h with high tidal volume.

To explore whether the observed effects of IL-17 on the p38 MAPK/MCP-1 pathway *in vitro* also occur *in vivo*, HTV ventilated mice were injected with anti-IL-17 antibody orrmIL-17 before ventilation. Anti-IL-17 antibody significantly reduced MCP-1 and p-p38 MAPK expression (**Figures 6A–C**), whereas rmIL-17 exerted the opposite effects (**Figures 6D, E**). At the same time, neither treatment affected the total level of p38 MAPK in lung or BALF (**Figures 6C, E**). These results confirm that IL-17 can upregulate MCP-1 by promoting the phosphorylation of p38 MAPK.

## Discussion

VILI is a pathophysiological process that leads to overwhelming inflammatory responses when alveolar macrophages, neutrophils, alveolar epithelial cells, and endothelial cells migrate to the lungs (27–29) and when pro-inflammatory cytokines are expressed. Current treatment methods for VILI are mainly based on regulating the tidal volume, oxygen concentration, and positive end-expiratory pressure of the ventilator (1, 30, 31). However, these therapies generally show low efficacy, highlighting the need to clarify the underlying mechanism of VILI in order to identify more effective targets.

In this study, we explored the role of IL-17 and the p38 MAPK/MCP-1 pathway in the pathogenesis of VILI. In our experiments *in vitro* and *in vivo*, HTV ventilation significantly increased the number of lung neutrophils, which secreted large amounts of IL-17, and this cytokine in turn upregulatedMCP-1 *via* the p38 MAPK pathway. Inhibition of IL-17 significantly reduced lung injury caused by HTV mechanical ventilation, suggesting that IL-17 may be a promising new target for the treatment of VILI-induced inflammation.

IL-17 receptors contain a conserved SEFIR domain, which binds to Act1 to activate downstream signal cascades (32, 33). Downstream targets of IL-17 include the NF-κB signaling pathway (34, 35), three MAPK pathways (JNK, ERK, and p38), and the phosphoinositide kinase pathway (36–38). Our findings about the role of IL-17 in VILI extend the list of conditions in which the cytokine can contribute to excessive inflammation, which include bacterial and fungal infections (39, 40). In fact, in certain cases of bacterial infection, higher levels of IL-17 may be beneficial to eliminate pathogens (41–43). Thus, whether elevated IL-17 levels lead to injury or benefit may depend on the cells secreting the cytokine and on other factors.

Inflammatory cells infiltrate into affected tissues, where they trigger local immune responses. Neutrophils, a type of polymorphonuclear leukocytes, are the first inflammatory cells recruited to inflammatory sites, where they help eliminate pathogens through multiple mechanisms (44–46). Although neutrophils are short-lived, they can be activated several times during inflammation (47, 48) and their chemotaxis is elevated in the lungs of patients with bacterial pneumonia or chronic bronchitis (49, 50). Here, we found a significantly high number of lung neutrophils in HTV ventilated mice, and flow cytometry revealed that lung neutrophils rather than Th17 cells are the main source of IL-17 during VILI. Therefore, we speculate that lung neutrophils may promote pneumonia response after HTV ventilation by promoting inflammation on their own, as well as by producing IL-17 to trigger inflammatory responses in other cell types. Future studies should clarify the role of neutrophils in VILI.

Although IL-17 participates in VILI through the p38 MAPK/MCP-1 pathway, there are still some limitations in this research that deserve improvement. First of all, HTV ventilation may cause respiratory alkalosis, we should continue to monitor and control the PCO2 and pH. However we had some difficulties in the monitoring methods mentioned above. Another limitation was there are some other factors that affect the severity of lung injury besides tidal volume. To reduce the influence of these factors, we should continuously monitor hemodynamics, PEEP or lung recruitment.

## Conclusion

Our study suggests that IL-17 produced by lung neutrophils can act *via* the p38 MAPK/MCP-1 pathway to drive pulmonary inflammatory responses in VILI. Inhibition of IL-17 can effectively relieve VILI, suggesting that IL-17 may serve as a new target for attenuating lung inflammation induced by HTV ventilation.

## Data Availability Statement

The datasets presented in this study can be found in online repositories. The names of the repository/repositories and accession number(s) can be found in the article/**Supplementary Material**.

## Ethics Statement

The animal study was reviewed and approved by The Animal Care & Welfare Committee of Guangxi Medical University.

## Author Contributions

LP designed and directed the overall study. XL wrote the manuscript and carried out experiments. WZ wrote the manuscript and guided the experiments. HD, RJ, and MY carried out the experiments. WG and SP collected and analyzed data. All authors read and approved the final manuscript.

## Funding

This work was supported by grants from the Natural Science Foundation of Guangxi (Grant No.2018GXNSFDA138007), the National Natural Science Foundation of China (Grant No. 81970078) and Innovation Project of Guangxi Graduate Education (Grant No.YCSW2021130).

## Conflict of Interest

The authors declare that the research was conducted in the absence of any commercial or financial relationships that could be construed as a potential conflict of interest.

## Publisher’s Note

All claims expressed in this article are solely those of the authors and do not necessarily represent those of their affiliated organizations, or those of the publisher, the editors and the reviewers. Any product that may be evaluated in this article, or claim that may be made by its manufacturer, is not guaranteed or endorsed by the publisher.

## Supplementary Material

Supplementary Figure 1 Lung neutrophils increase after HTV. Neutrophils in mouse lung cells. Data are shown as mean ± SD (n = 6). **P* < 0.05 vs. CON group, ^#^ *P* < 0.05 vs. H0.5h group. CON, non-ventilated mice; H0.5h, H0.75h, H1h, H4h, ventilated for 0.5h, 0.75h, 1h and 4 h with high tidal volume.
